# Composite Materials Based on Polymeric Fibers Doped with Magnetic Nanoparticles: Synthesis, Properties and Applications

**DOI:** 10.3390/nano12132240

**Published:** 2022-06-29

**Authors:** Ioan Bica

**Affiliations:** Faculty of Physics, West University of Timisoara, 300223 Timisoara, Romania; ioan.bica@e-uvt.ro

The increasingly sophisticated requirements of contemporary society, in relation to the assessment of environmental and health factors, are receiving much attention from the scientific community. There are many activities regarding the production of fabrics based on composite materials, such as temperature sensors or pH sensors, with functions of monitoring environmental factors and health [1,2,3,4,5,6,7,8,9] or in magnetic field protection of implant holders and pacemakers [9,10].

In the efforts made by the academic community to satisfy these requirements, an important role is played, as will be shown below, by composite materials based on natural, artificial polymeric fibers or their combinations, and magnetizable nano-microparticles. In the complex form known as composite materials, polymeric fibers have the role of support. The determining role is played by the magnetizable phase. The latter may be in the form of iron nanoparticles [11,12], colloidal magnetic nanoparticles [13,14], octopus-shaped magnetizable microparticles [15], iron microtubes [16], iron microspheres and/or iron pore microspheres [17,18] and iron oxide nano-microfibers [19], generated by plasma processes. Their size and shape are controlled by the electro-thermal parameters of the plasma and by the quantities of thermally processed materials in inert or oxidizing gas environments [11,12,13,14,15,16,17,18,19]. From liquid solutions, based on the nano–microfibers of iron oxides and silicone oil, magnetorheological suspensions are obtained whose electrical properties are useful in making magnetically active composites [20].

Cotton fiber composites [21] are made of microfibers as described in Refs. [19,20] and a well-specified amount of carbonyl iron microparticles and silicone oil. The absorption coefficient of the medium-frequency electric field energy is fixed by the value of the microfiber mass fraction and is significantly modified by the applied magnetic field [21]. By electrostatic interaction to the cotton fibers of well-chosen quantities of mixtures composed of barium titanate nanoparticles and carbonyl iron microparticles, composites with remarkable physical properties are obtained [22]. When fixed between two copper plates and the assembly is consolidated in a silicone rubber mantle, magneto-tactile transducers are obtained.

Following this research direction, in Ref. [23] the composites, made of cotton microfibers with carbonyl iron microparticles, are distinguished by dielectric properties controlled by a static magnetic field, a static electric field and combinations thereof. The electrical devices made with these composites can be useful in recording the limits allowed by the labor protection rules for magnetic and electric fields. In commercial sponges, carbonyl iron microparticles attach electrostatically to polyurethane fibers, forming a magnetizable composite [24]. Inserted between copper cylindrical electrodes, they form a capacitor. Using the planar capacitor method [25], the authors of the paper [24] obtain information on the modification of the dispersion coefficients and of the energy absorption of a medium-frequency electromagnetic field, and respectively when applying a static magnetic field. The composite obtained in Ref. [24] is low-cost and useful for the absorption of medium frequency electromagnetic radiation. The electrical device based on this composite stands out as an excellent electromagnetic radiation sensor.

It is known that white light [26,27], magnetic field [28,29], low- and medium-frequency electromagnetic field [30,31,32], carbonyl iron microparticles [33], honey [34,35] and turmeric powder are good remedies [28], on a case-by-case basis, in medical treatments. Based on the reports published in Refs. [26,27,28,29,30,31,32,33,34,35], composite membranes based on cotton fabric, with square mesh, having a side of 3 mm size, honey and carbonyl iron microparticles are manufactured, as shown in Refs. [36,37]. By adjusting the intensity of the static magnetic field, superimposed over the medium-frequency electromagnetic field, the white light transmission changes and the value of the dielectric loss coefficient is adjusted. Through this procedure, the thermal transfer of well-chosen components from bee honey, or the controlled lighting of the incident surfaces based on medical prescriptions can be achieved.

Investigations in static magnetic fields superimposed on an alternating medium-frequency electric field of composites based on bee honey and turmeric powder is the subject of Refs. [38,39]. In these works, the preparation of the composites is described, and it is shown that for well-specified amounts of turmeric powder and well-chosen frequencies of the medium frequency field, one can achieve those values of the tangent of the dielectric loss angle which allows the thermal transport of the substance, from the components of the three-phase mixture. The device made in Refs. [38,39] can be assimilated to a medical device. Similar results are reported in Ref. [40], where cotton fabric with bee honey and iron microparticles are introduced in beeswax. Here, the kinetics of the crystallization of the glucose solution in the magnetic field is studied, with possible applications in the study of the crystallization of the glucose solutions and in the realization of medical devices. By preparing cotton fabric with $\gamma -Fe_{2}O_{3}$ nanoparticles, magnetoresistors are obtained in which the resistive function is magnetically controlled [41]. An electrical device, with capacitive, resistive and electric voltage generation functions, is made in Ref. [42]. The electrical device consists of two copper textolite plates between which there are composites based on cotton fibers with barium titanate nanoparticles and carbonyl iron microparticles. In a magnetic field, the capacitive, resistive and piezoelectric functions of the device are magnetically controlled. For shielding devices significantly influenced by electromagnetic interference, in Ref. [43], membranes are manufactured that have polyurethane fibers and magnetite nanoparticles in their composition. The membranes are thin (0.45 mm), flexible and offer a good shield against the electromagnetic field.

In short, we consider that this Special Issue, although it only has five works, opens a research direction aiming at the realization of magnetically and electrically active composites, based on polymeric fiber fabrics, which is useful for the realization of necessary means for vital parameter control, notification and protection of the human being from unfriendly environmental factors.

## Data Availability

Not applicable.
